# Eosinophilic angiocentric fibrosis: A case report and a review of the literature

**DOI:** 10.1016/j.ijscr.2025.111433

**Published:** 2025-05-13

**Authors:** Jood K. Alotaibi, Raghad A. Alkhaldi, Naif H. Alotaibi, R.M. Mumtaz, M. Anas Dababo

**Affiliations:** a College of Medicine, Imam Abdulrahman Bin Faisal University, Dammam, Saudi Arabia; b College of Medicine, Imam Mohammad Ibn Saud Islamic University, Riyadh, Saudi Arabia; c Department of Otolaryngology, Head and Neck Surgery, King Faisal Specialist Hospital and Research Centre, Riyadh, Saudi Arabia; d Department of Otolaryngology, Head and Neck Surgery, King Faisal Specialist Hospital and Research Centre, Riyadh, Saudi Arabia; e Department of Pathology and Laboratory Medicine, King Faisal Specialist Hospital and Research Centre (KFSHRC), Riyadh, Saudi Arabia

**Keywords:** Eosinophilic Angiocentric Fibrosis, Case report, IgG-4-related disease

## Abstract

**Background:**

Eosinophilic Angiocentric Fibrosis (EAF) is a rare inflammatory condition affecting the upper respiratory and sinonasal tracts. This study aims to elaborate on the clinical and pathological features of a patient with extensive, rare EAF involving the sinonasal tract, subglottic region, and orbit.

**Case presentation:**

A fifty-five-year-old female patient with a known case of bronchial asthma, hypothyroidism, and IgG-4-related necrotizing blepharitis presented to our clinic with a 14-year history of bilateral nasal obstruction and post-nasal drip despite medical and surgical intervention. Rhinoscopy revealed fibrosis and adhesion of the nasal cavity and nasopharynx, along with grade I subglottic stenosis. Functional endoscopic sinus surgery was performed to relieve her symptoms and obtain a specimen confirming the diagnosis of EAF.

**Discussion:**

Given the wide variety of differential diagnoses for bilateral nasal obstruction, imaging and histopathological evaluation are necessary to diagnose EAF. The characteristic onion-skin pattern reflecting the perivascular eosinophil-rich inflammatory infiltrate with subsequent concentric fibrosis is the gold standard in diagnosing these patients.

**Conclusion:**

Despite advancements in clinical practice, a clear management guideline for patients with EAF has not yet been established. In our paper, we pool our management approach with the literature.

## Highlights

• Eosinophilic Angiocentric Fibrosis (EAF) is a rare inflammatory condition affecting the upper respiratory tract and paranasal sinuses.
• EAF has a pathognomic onion-skin pattern, reflecting the perivascular eosinophils-rich inflammation with concentric fibrosis.
• Symptoms of EAF include nasal obstruction, facial pain, post-nasal drip, and epistaxis; a thorough diagnostic approach is essential.
• EAF can be managed medically or surgically; however, no clear guidelines exist, leading to a personalized patient-oriented decisions.

## 1 Introduction

Eosinophilic Angiocentric Fibrosis (EAF) is a rare fibrotic condition first described by Holmes and Panje in 1983 as “Intranasal granuloma faciale” [1]. The term was then redefined in 1985 by Robert and McCann, as a mucosal lesion accompanied by thickening of the submucosal connective tissue [2,3]. It is a benign disease of an unknown etiology that affects the upper respiratory tract, mainly the paranasal sinuses and nasal septum; rarely, it involves the subglottic region and orbit [2]. This condition requires a high clinical suspicion, supportive imaging modalities, and laboratory testing to establish the diagnosis. Most importantly, its definitive diagnosis is achieved by histopathological evaluation, which shows the pathognomonic onionskin pattern reflecting the perivascular eosinophils-rich inflammatory infiltration with subsequent concentric fibrosis [4]. Sinonasal EAF typically presents with prolonged nasal obstruction, epistaxis, nasal deformities, facial pain, and epiphor [1]. This paper presents a case with clinicopathological findings of EAF involving the sinonasal tract, subglottic region, and orbit. This paper has been reported following the updated consensus of the Surgical Case Report (SCARE) guidelines [5].

## 2 Case presentation

**Fig. 1:**
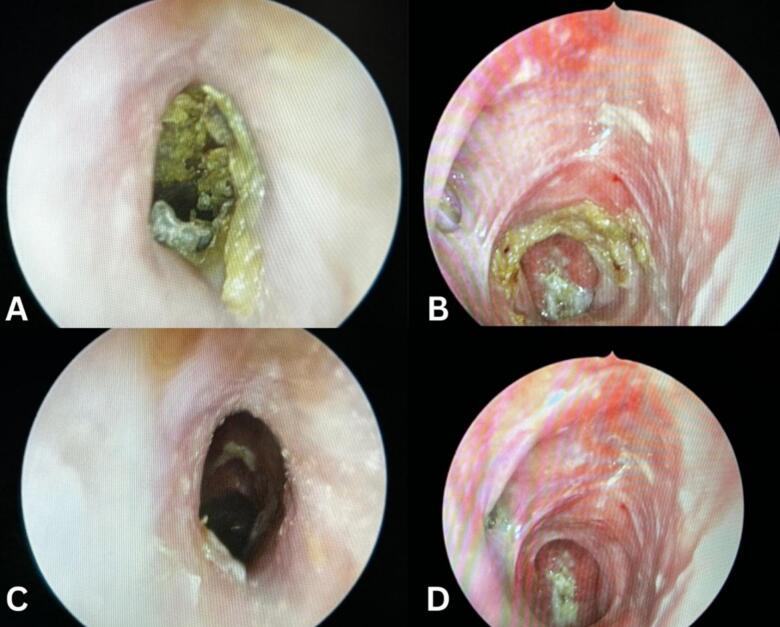
Post-operative endoscopic findings of the nasal cavity, showing nasal mucosal crusting within the vestibule (A) and the nasopharynx (B), narrowed nasal vestibule (C), adhesions and fibrosis of the nasal cavity and nasopharynx (D).

**Fig. 2:**
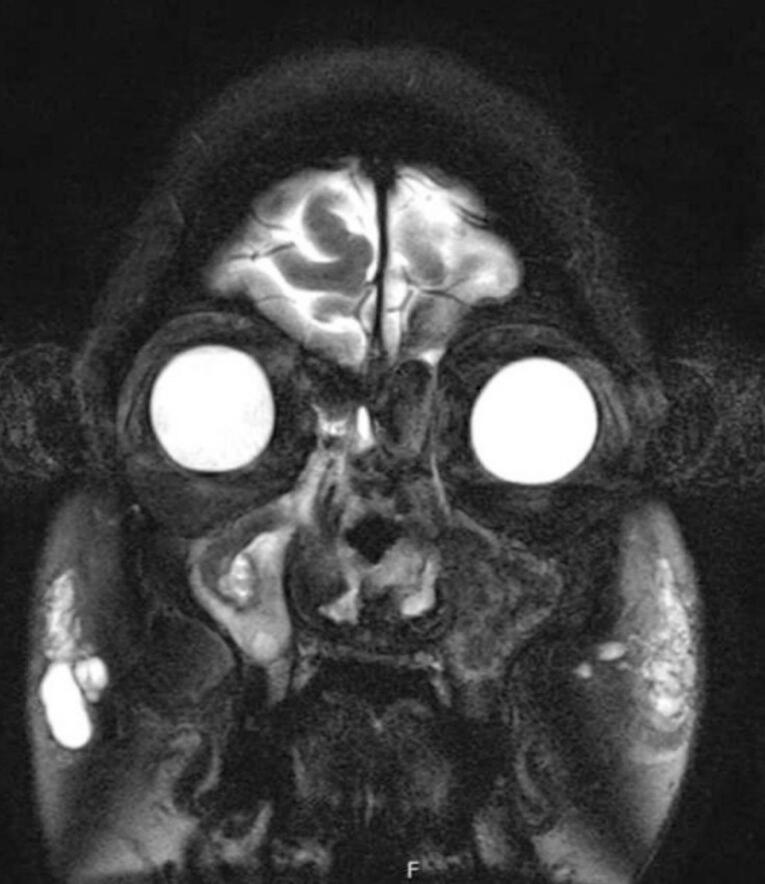
Preoperative coronal T2-weighted MRI of the paranasal sinuses showing an opacification within the bilateral nasal cavities, left maxillary, ethmoid, and sphenoid sinuses.

We present a case of a fifty-five-year-old female patient known for controlled bronchial asthma, hypothyroidism, and IgG-4-related necrotizing blepharitis. The patient is a non-smoker, non-alcoholic, and retired from work. She was referred to our otolaryngology tertiary clinic due to her refractory nasal symptoms. She presented with a 14-year history of recurring bilateral nasal obstruction, mainly on the right side, associated with post-nasal drip, facial pressure, and hyposmia. However, she denies mucopurulent nasal discharge and visual disturbance, and her systemic review is unremarkable. Nonetheless, an allergic reaction was documented following her ingestion of hazelnuts and nonsteroidal anti-inflammatory drugs (NSAIDs). The patient reported that her symptoms were temporarily managed at another institution using intranasal steroid sprays. However, her condition reached a stage where more invasive intervention was needed, as she underwent three Functional Endoscopic Sinus Surgeries (FESS). Yet, her nasal symptoms were refractory. Upon physical examination, rhinoscopy revealed bilateral small nasal vestibules associated with fibrosis and adhesion of the nasal cavity and nasopharynx, nasal crusting (Fig. 1), and grade I subglottic stenosis evident by fiberoptic nasoendoscopy with an approximate luminal obstruction of 50 %, possibly attributed to the underlying EAF. However, a biopsy was not taken from this lesion. A Magnetic Resonance Imaging (MRI) of the paranasal sinuses showed an opacification within the bilateral nasal cavities, left maxillary, ethmoid, and sphenoid sinuses (Fig. 2).

**Fig. 3:**
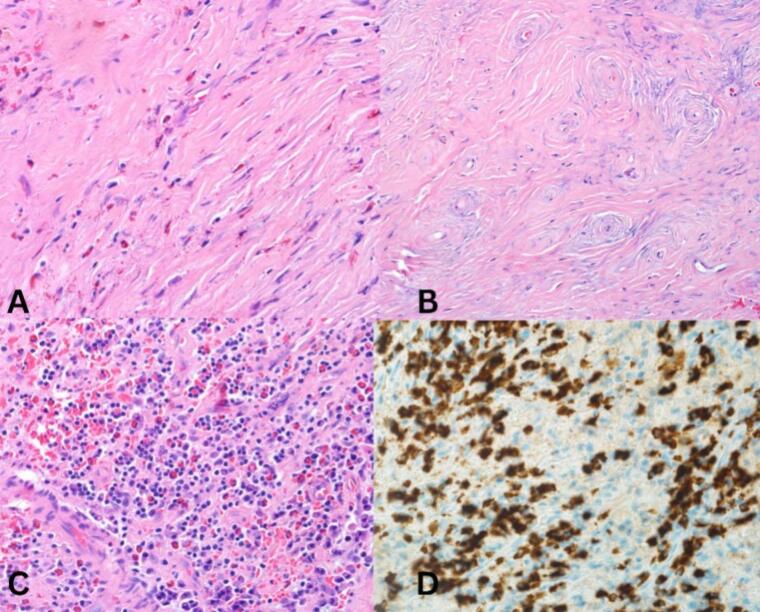
: Histopathological findings of nasal mass biopsy, showing 40× dense fibrosis with eosinophils (A), 40× onion skin fibrosis around blood vessels (B), 20× showing many plasma cells and eosinophils (C), and IgG4 immunostaining shows numerous IgG4+ plasms cells over 100 in high power (D), Thus, confirms the diagnosis of EAF IgG4-related disease.

Accordingly, a revision FESS was decided to improve the patient's symptoms and obtain a specimen from the left nasal mucosa and the frontal sinus cyst for histopathological evaluation. Specimens collected during the surgery revealed evidence of left nasal mucosal ulceration and purulent acute inflammation. Additionally, there was psudocystic formation involving the frontal sinus, which was lined by histiocytes in a background of dense fibrosis (Fig. 3). These findings, along with her clinical picture, supported a diagnosis of EAF. The patient had an uneventful postoperative course and was given a lifelong weekly follow-up appointment for her nasal crustations to be removed to improve her quality of life.

## 3 Discussion

EAF is an inflammatory process that develops slowly and progressively in its course, with a predilection for the upper respiratory and sinonasal tract. Rarely does it involve the subglottic region and orbit [6]. In our case, the patient was not only experiencing sinonasal involvement; rather, she had IgG-4-related necrotizing blepharitis and grade I subglottic stenosis. Histopathologically, EAF is characterized by the presence of multiple scattered eosinophils in a background of dense fibrosis [6]. The possible etiological theories of EAF include a past personal history of granuloma faciale, hypersensitivity to pollen, dust mites, animal dander, and certain foods due to high Immunoglobulin-E levels, and previous traumatic or iatrogenic injury to the sinonasal tract [1]. In 2011, Deshpande et al. suggested that EAF lies under the spectrum of immunoglobulin-G4-related disease, necessitating a modified approach to patients with this condition [6]. Nevertheless, not all cases of EAF fit the diagnostic criteria of immunoglobulinG4-related disease.

The majority of patients with EAF are females, presenting with non-specific signs and symptoms and usually delayed diagnosis [7]. These signs and symptoms include nasal obstruction, epistaxis, nasal deformity, facial pain, epiphora, and proptosis that collectively progress slowly over time [7]. Computed Tomography (CT) and MRI are non-specific diagnostic tools; however, the presence of an opacification involving the nasal cavity and paranasal sinuses aids in the diagnosis [1]. Thus, a definitive histopathological diagnosis is required to establish the diagnosis of EAF, which will show the pathognomonic onion-skin appearance of perivascular fibrosis that is associated with eosinophilic infiltration [7].

Based on our survey of the relevant scholarly literature, we have identified sixty-two cases of sinonasal EAF, aged twelve to eighty-one years. The median age among these patients was forty-nine years. The data revealed a relative female preponderance with a female: male ratio of 1.13: 1, equivalent to thirty-three female patients, while the remaining were male. The literature provides adequate evidence that the most frequently reported symptoms comprise nasal obstruction, Post-Nasal Drip (PND), mucosal crusting, and epistaxis. More overt manifestations, such as noticeable nasal deformity, are also commonly reported. Our patient experienced all these symptoms except for epistaxis (Table 1).

**Table 1:** Summary of reported cases of eosinophilic angiocentric fibrosis.

Reference	Age	Gender	Clinical Presentation	Topography	Management
Roberts PF et al. [1]	27 years-old	Female	Bilateral nasal obstruction	Nasal mucosa	Systematic antihistamine, systematic and local steroids, and septoturbinoplasty.
	33 years-old	Female	Exertional shortness of breath	Subglottic area	Tracheostomy tube followed by laryngotracheoplasty.
	59 years-old	Female	Bilateral nasal obstruction	Nasal mucosa and skin of the nose	Antihistamine and nose exploration.
			Urticaria		
			Painful red patch over the nasal bridge		
Karligkiotis et al. [1]	46 years-old	Male	Proptosis with lateral globe displacement	Right orbit into anterior ethmoid	Endoscopic diagnostic debulking.
			Right-sided nasal obstruction		
			Supra-orbital headache		
WJ Kim et al. [1]	29 years-old	Female	Nasal obstruction	Posterior wall of right maxillary sinus	Systematic steroids, Fluticasone-salmeterol, docyfylline, mucolytics agents for 3 months followed by surgical resection.
			Productive cough		
			Dyspnea		
Keratli H et al. [1]	30 years-old	Female	Mass under the right eye associated with epiphora	Medial orbit, extending to the anterior ethmoid sinuses and right middle turbinate.	Debulking followed by systametic steroids and antihistamine.
Leibovitch I et al. [1]	61 years-old	Male	Right peri-orbital edema	Right medial orbital wall	Systemic steroids and regular follow up.
			Right painful proptosis		
Deshpande V et al. [1]	63 years-old	Male	Ocular tearing	Nasal and lacrimal glands	Systemic steroids and surgical excision.
			Nasal congestion		
				Lung	
	81 years-old	Female	N/A	Left nasal mucosa	
	31 years-old	Female	N/A	Orbit	
				Maxillary and ethmoid sinuses	
				Nasal mucosa	
	54 years-old	Female	N/A	Right lacrimal gland	
	55 years-old	Male	N/A	Orbit	
Nguyen DB et al. [1]	45 years-old	Male	Bilateral nasal obstruction	Bilateral nasal bones	Surgical excision
				Nasal septum	
Jain R et al. [1]	31 years-old	Female	Right epiphora	Left maxillary and ethmoid sinuses	Systemic steroids and surgical excision
			Left orbital mass		
				Orbit	
	57 years-old	Male	Nasal congestion	Balateral lateral nasal walls	
			Epiphora		
			Proptosis	Lacrimal gland	
			Hyposmia		
	27 years-old	Female	Nasal obstruction	Bilateral lateral nasal walls	
	51 years-old	Female	Nasal mass	Right lateral nasal walls	
			Nasal obstruction		
			Epiphora		
Li Y et al. [1]	27 years-old	Female	Nasal obstruction	Anterior nasal cavity	Endoscopic surgery and long-term intranasal topical corticosteroids
			Nasal discharge		
			Epistaxis		
			Facial pain		
			Chronic headache		
	12 years-old	Female	Epistaxis	The anterior septum of the right nasal cavity	Endoscopic excision
Narayan J et al. [1]	72 years-old	Female	Complete stenosis of the left nostril	Nasal septum	Medical treatment
				Left lateral nasal wall	
			Nasal swelling		
Paun S et al. [1]	37 years-old	Female	Epistaxis	Left nasal cavity	Surgical management, oral steroids, and azathioprine
			Epiphora		
			Symptoms of eustachian tube dysfunction		
	68 years-old	Male	Nasal obstruction	Nasal bridge	Surgical management
				Forehead	
	57 years-old	Female	Nasal swelling	Internal and external structure of the nose	Dapsone, hydroxychloroquine, azathioprine, and oral steroids
	58 years-old	Female	N/A	Lateral nasal wall	Surgical management
Nigar E et al. [1]	67 years-old	Female	Nasal obstruction	Right nose	Medical management
			Purple discoloration of the overlying skin		
Pareira EM et al. [1]	52 years-old	Male	Nasal obstruction	Nasal mucosa and septum	Surgical management
			Left -sided tinnitus and hearing loss		
				Facial sinus bones	
			Nasal pruritus		
			Sneezing		
Slovik Y et al. [1]	45 years-old	Male	Nasal obstruction	Nasal bones, septum, and skin	Vasoconstrictive nasal drops as needed
			Hyponasal speech		
			Cosmotic deformity	Right maxillary and ethmoid sinuses	
Loane J et al. [1]	42 years-old	Male	Nasal obstruction	Nasal mucosa	Surgical and medical management
			Post-nasal drip		
Yung A et al. [1]	66 years-old	Female	Recurrent epistaxis	Nasal mucosa	Medical management
				Right preauricular skin lesion	
	45 years-old	Female	Nasal obstruction	Nasal mucosa and skin	
			Recurrent epistaxis		
Clauser L et al. [1]	31 years-old	Male	Nasal obstruction	Nasal cavity and septum	Surgical management
			Nasal pain		
			Epistaxis		
Kosarac et al. [1,9]	19 years-old	Female	Nasal congestion	Right maxillary sinus	Surgical excision
			Facial pain		
	31 years-old	Female	Nasal obstruction	Right nasal cavity	
	49 years-old	Male	Nasal obstruction	Nasal septum	
Matai V et al. [1]	51 years-old	Male	Nasal obstruction	Nasal septum mucosa	Medical and surgical management
			Snoring		
Thompson LD et al. [1]	28 years-old	Male	Nasal obstruction	Left maxillary sinus and nasal septum	Surgical excision and systemic corticosteroids
			Epistaxis		
	49 years-old	Female	Nasal obstruction	Nasal septum	Septoturbinoplasty and systemic corticosteroids
	64 years-old	Female	Nasal obstruction	Right maxillary sinus and nasal septum	Surgical excision and systemic corticosteroids
Yavuzer R et al. [1]	65 years-old	Female	Chronic sinusitis	Nasal mucosa and septum	Surgical management
Goldman NC et al. [1]	50 years-old	Female	Nasal obstruction	Nasal mucosa	Surgical and medical management
Holme et al. [1]	72 years-old	Female	Nasal obstruction	Nasal mucosa	Pulsed dye laser, dapsone and clofazimine
Onder S et al. [14]	45 years-old	Male	Nasal obstruction	Nasal septum	Surgical and medical management
Chinelli PA et al. [1]	31 years-old	Female	Nasal obstruction	Nasal mucosa	Medical management
Owa AO et al. [1]	41 years-old	Male	Nasal obstruction	Nasal septum	Surgical and medical management
Sunde J et al. [1]	63 years-old	Male	Nasal obstruction	Nasal septum	Medical management
			Hyposmia		
Tabaee A et el. [1]	79 years-old	Male	Nasal congestion	Nasal septum and lateral wall	Surgical management
			Nasal tip tenderness		
			Nasal swelling		
Yang BT et al. [1]	26 years-old	Female	Nasal obstruction	Nasal septum	Surgical and medical management
			Epistaxis		
			Eyelid swelling		
			Proptosis		
	16 years-old	Male		Nasal septum	
	62 years-old	Female		Right lateral nasal wall	
	28 years-old	Male		Nasal septum	
	24 years-old	Female		Nasal septum	
	73 years-old	Male		Left lateral nasal wall	
Burns BV et al. [1]	38 years-old	Male	Nasal obstruction	Right nasal mucosa	Surgical and medical management
			Nasal swelling		
Holmes DK et al. [1]	49 years-old	Male	Nasal obstruction	Right nasal mucosa	Medical management
Valenzuela AA et al. [1]	43 years-old	Male	Nasal obstruction	Nasal cavity	Regular follow-ups
			Bilateral epiphora		
Watanabe N et al. [1]	51 years-old	Male	Nasal obstruction	Nasal septum	Surgical management
				Ethmoid bone	
Gorostis et al. [3]	61 years-old	Male	Headache	Right ethmoido-orbital region	Surgical and medical management
			Nasal obstruction		
			Exophthalmos		
			Blindness		
Javadirad et al. [2]	60 years-old	Female	Nasal obstruction	Nasal septum	Surgical management
			Post-nasal drip		
Sazgar AA et al. [4]	45 years-old	Male	Nasal obstruction	Anterior nasal cavity	Surgical management
			Nasal discharge	Right maxillary alveolar ridge	
			Epistaxis		
			Post-nasal drip		
Farina J et al. [15]	76 years-old	Male	N/A	Nasal mucosa	Surgical management
Saenz Ibarra B et al. [16]	37 years-old	Female	Facial pain	Nasal mucosa	Surgical and medical management
			Chronic sinusitis		
			Allergic rhinitis		
			Nasal obstruction		
Legare et al. [17]	58 years-old	Male	Left eyelid swelling	Nasal cavity	Medical management
			Epistaxis	Lungs	
				Medial periorbital soft tissue	

Despite extensive documentation of this rare condition, the treatment guidelines for EAF remain unclear [9,10]. Previous cases have reported various management options, including surgical excision, FESS, and the use of intranasal or systemic corticosteroids [2]. Surgical excision is typically performed in advanced cases but is associated with a high recurrence rate. The use of intranasal or oral steroids has shown limited improvement [2]. Rituximab may benefit patients with refractory symptoms in both medical and surgical treatment [8].

In our case, the patient experienced symptomatic relief following revision FESS and three doses of Rituximab. However, she requires ongoing suctioning to manage nasal mucosal crusting. This ambiguous treatment approach presents a clinical dilemma regarding the optimal management of EAF patients, especially in patients like ours with rare extensive involvement in her sinonasal tract, subglottic region, and orbit.

## Consent

Written informed consent was obtained from the patient for publication and any accompanying images. A copy of the written consent form is available for review by the editor-in-chief of this journal upon request.

## Ethical approval

The ethical committee has approved this case report.

## Guarantor

Jood K. Alotaibi.

## Funding

This paper has not received any funding.

## Author contribution

Jood K. Alotaibi, Raghad Alkhaldi, and Ramiz Muhammed Mumtaz have constructed and write the introduction, reviewed the literature for similar and studies, as well as the conclusion.

Naif Alotaibi contributed to the paper by writing the case itself, providing the supplementary imaging.

Anas Dababo provided the histopathological slides, and reported the histopathological evaluation of the patient.

## Declaration of competing interest

There is nothing to disclose.
